# A mindfulness-based stress prevention training for medical students (MediMind): study protocol for a randomized controlled trial

**DOI:** 10.1186/s13063-014-0533-9

**Published:** 2015-02-08

**Authors:** Sophie Merle Kuhlmann, Arne Bürger, Günter Esser, Florian Hammerle

**Affiliations:** Department for Child and Adolescent Psychiatry and Psychotherapy, University Medical Center of the Johannes Gutenberg University Mainz, Langenbeckstraße 1, 55131 Mainz, Germany; Department of Psychology, University of Potsdam, Karl-Liebknecht-Straße 24/25, 14476 Potsdam, Germany

## Abstract

**Background:**

Medical training is very demanding and associated with a high prevalence of psychological distress. Compared to the general population, medical students are at a greater risk of developing a psychological disorder. Various attempts of stress management training in medical school have achieved positive results on minimizing psychological distress; however, there are often limitations. Therefore, the use of a rigorous scientific method is needed. The present study protocol describes a randomized controlled trial to examine the effectiveness of a specifically developed mindfulness-based stress prevention training for medical students that includes selected elements of cognitive behavioral strategies (MediMind).

**Methods/Design:**

This study protocol presents a prospective randomized controlled trial, involving four assessment time points: baseline, post-intervention, one-year follow-up and five-year follow-up. The aims include evaluating the effect on stress, coping, psychological morbidity and personality traits with validated measures. Participants are allocated randomly to one of three conditions: MediMind, Autogenic Training or control group. Eligible participants are medical or dental students in the second or eighth semester of a German university. They form a population of approximately 420 students in each academic term. A final total sample size of 126 (at five-year follow-up) is targeted. The trainings (MediMind and Autogenic Training) comprise five weekly sessions lasting 90 minutes each. MediMind will be offered to participants of the control group once the five-year follow-up is completed. The allotment is randomized with a stratified allocation ratio by course of studies, semester, and gender. After descriptive statistics have been evaluated, inferential statistical analysis will be carried out with a repeated measures ANOVA-design with interactions between time and group. Effect sizes will be calculated using partial η-square values.

**Discussion:**

Potential limitations of this study are voluntary participation and the risk of attrition, especially concerning participants that are allocated to the control group. Strengths are the study design, namely random allocation, follow-up assessment, the use of control groups and inclusion of participants at different stages of medical training with the possibility of differential analysis.

**Trial registration:**

This trial is recorded at German Clinical Trials Register under the number DRKS00005354 (08 November 2013).

## Background

### Stress and psychological morbidity in medical school

Scientific research indicates that medical training is associated with a high prevalence of psychological distress. The occurrence of burnout symptoms among medical students is reported to range from 45% to 71% [1], and numerous data suggest an increase in psychological morbidity. This becomes evident in a higher symptom load for mental disorders when comparing medical students with an age-matched control sample. Medical students at different stages of their training scored significantly higher on scales of the Patient Health Questionnaire (PHQ) such as ‘major depressive syndrome’ or ‘other anxiety syndromes’ [2]. A poorer mental health, measured by the ‘Short Form-12 Health Survey’ (SF-12), is also reported for medical students in their first, second and third year of studies compared to a reference sample [3]. These findings of a high level of overall psychological distress in medical students is confirmed by a variety of scientific studies and summarized in a review of the literature [4].

Studies providing data that enable an assessment of the psychological morbidity in medical training in contrast to other courses of studies are scarce and results differ. Aktekin *et al*. [5], for example, revealed that with the General Health Questionnaire (GHQ), a higher percentage of medical students scored above threshold in comparison to students of economics and physical education. However, according to Dahlin *et al*. [6], the prevalence of mental health problems in need of treatment and the frequency of ‘help seeking’ did not differ between medical and business students. More research is needed investigate whether medical students experience more stress than other high-achieving student populations.

Nevertheless, a high level of perceived medical school stress is strongly associated with psychological problems [7-9]. For example, high ratings on a stress inventory within a cohort of medical students are positively correlated with depressive symptoms [10]. Consequently, there is a demanding risk of developing mental health problems in later professional life [11]. The competency and professionalism of physicians may be affected by poor mental health, and consequently, the treatment of patients may be impaired [12]. Therefore, health promotion and prevention programs of psychological symptoms during medical school seem necessary and beneficial for both physicians and patients.

### Intervention programs

In a review of the literature, Shapiro *et al*. [13] discovered over 600 articles discussing the need to address the stress of medical education. However, only 24 studies reported intervention programs. A decade later, the number of stress-management programs has not increased significantly [14,15], and to our knowledge, no study of a prevention program in German medical schools has yet been published. There has been a variety of stress-management programs offered in medical schools aimed at relaxation training, mindfulness-based stress reduction, self-hypnosis, educational discussion groups on self-care, support groups, mentoring programs and others [16-21]. A review of the literature makes it apparent that intervention programs are helpful [22], and the students who completed the trainings were in favor of the programs being offered regularly [13]. This impression is confirmed by Yusoff *et al*. [14], who reviewed 23 studies of stress-management programs. Regardless of the duration of training, they all reported positive outcomes on several areas related to health, such as improved psychological health, quality of life or increased awareness of stress and stress management. Despite achieving good effects, the studies are reported to have limitations, and the necessity of using rigorous scientific method is needed [13]. According to Yusoff *et al*. [14], only one in 23 studies used a random sampling method in selecting participants, 13 studies had control groups and only seven randomly assigned participants to control and intervention groups. Furthermore, follow-up assessments to evaluate the prevention effects of the interventions are mostly missing or the intervals are short [13-15].

In order to address these limitations, we use a randomized controlled trial that implies an allocation of the participants to three groups (experimental treatment, standard treatment and control without treatment). Given that nothing is known about the most efficient point in time to offer stress-management training, we examine medical students at two different stages of medical training. Additionally, we expect our research design to enable us to determine which of the two interventions work best for whom.

In order to address the psychological health of medical students, we have developed a mindfulness-based stress prevention training tailored to the needs of students in medical education. Following the concept of dialectical behavior therapy developed by Linehan [23], we combine acceptance strategies as described below (concept of mindfulness) with change strategies (contents of cognitive behavioral therapies). This combination enables the students to react to stressful conditions by modifying the situation, adapting their judgement mechanisms, or otherwise to meet these conditions with acceptance. Therefore, the development of the training is based on scientifically proven concepts of stress reduction like Kabat-Zinn [24], Lehrhaupt and Meibert [25], Hassed [26], Linehan [27] (German adaption, Bohus and Wolf [28]) and Kaluza [29].

### Concept of mindfulness and proof of efficacy

Mindfulness is an ancient Buddhist practice that became popular in the Western culture by the work of Jon Kabat-Zinn. It is characterized by ‘paying attention in a particular way: on purpose, in the present moment, and nonjudgementally’. [24]. Mindfulness therefore includes ‘paying attention to our thoughts and emotions in the present moment’ [30] and changing our attitudes towards them. This is of high relevance, given that perception of stress often originates in stressful thoughts about the future or ruminations about the past [30]. The integration of this capacity of nonevaluative moment-to-moment awareness into everyday life may function as a coping resource for dealing with difficult emotions [24] and the experience of stress. Mindfulness-based stress reduction (MBSR) is a group-intervention program developed by Jon Kabat-Zinn, which has been proposed as an approach to address a wide spectrum of clinical populations as well as nonclinical groups. The success of this approach becomes apparent in a meta-analysis that showed medium effect size of 0.5, including 20 reports of controlled and observational investigations [31].

In addition, mindfulness practice has already been successfully employed in medical school [16,17,21,32,33]. The existing data show good results from a before and after comparison concerning improvement on scales such as the perception of stress, depression, anxiety or quality of life. As mentioned above, follow-up data to evaluate the prevention effects is largely absent; only Warnecke *et al*. [32] found that the positive effect of mindfulness treatment was maintained for 8 weeks post-trial. Therefore, our investigation aims at indicating significant changes even after one year post-trial.

Positive effects of mindfulness-oriented interventions on psychological health of clinical and nonclinical samples have been repeatedly confirmed [34,35]. There seems to be a wide variety of health promoting aspects due to mindfulness training beyond the results already mentioned. For example, empirical literature indicates positive changes in psychological constructs such as ‘self as a source of control’ [36] or ‘satisfaction with life’ [37]. In contrast to relaxation training, mindfulness meditation seems to be specifically effective in reducing distress by positively influencing distractive and ruminative thoughts and behavior [21]. As rumination has been considered a risk factor for a number of psychological disorders [38], mindfulness training gains importance as a prevention strategy leading to a reduction in rumination by increasing metacognitive awareness [39]. Furthermore, correlation studies refer to associations between trait mindfulness and aspects of psychological health such as self-esteem [40], empathy [41], sense of autonomy [42] or optimism [42].

### Risk factors

Before developing our stress prevention training, we reviewed literature to obtain information regarding risk factors associated with psychological morbidity in medical education. We intended to develop a program based on findings of specific risk factors to increase its prevention effect and to affect the special needs of medical students. Unfortunately, the number of prospective studies is limited and often the results have not been confirmed. In addition, limitations are inherent in the research design or statistical analysis, for example, no multivariate aspects. The existing empirical literature indicates that deterioration of psychological health of medical students is associated with some of the following factors: study related stressors [9], exposure to life events [8,43], personality traits such as impulsivity [44], maladaptive perfectionism [45], external locus of control [46], performance-based self-esteem [44] and coping mechanisms such as wishful thinking [46]. Scores were generally low in active coping [9] or avoidant coping strategies [47]. In a longitudinal investigation [48,49], self-criticism as a personality variable was discovered to be a strong predictor of stress symptoms over a period of 10 years. Medical students were repeatedly examined from their fourth year of study up to the time when they were working as general practitioners. Remarkably, the hours worked in the past week did not significantly correlate with current stress levels, but the degree of self-criticism as students did.

In order to gain information on whether stress prevention training is able to influence these risk factors, we will obtain these factors by psychometric measures.

### Primary and secondary aim

The primary aim of this research is to examine the effectiveness of a mindfulness-based stress prevention training for medical students (MediMind). It is hypothesized that MediMind will be more effective in the reduction of stress and prevention of psychological morbidity as a standard treatment and more effective than a control condition. The evaluation will be based on an interaction-related stress concept [50], in which stress is defined as a nonmatch between requirements and the resources available to a person according to the transactional model of stress and coping by Lazarus [51]. Therefore, we expect MediMind to have a beneficial effect on the experience of stress and coping strategies as primary and co-primary outcome. Furthermore, as secondary outcome we expect MediMind to have a preventive effect on psychological morbidity that will be visible only after reviewing the follow-up surveys.

The secondary aim of this research is to examine the effect of MediMind on risk factors associated with psychological morbidity in medical students compared to a standard treatment and a control condition. It is expected that MediMind has a beneficial effect on rumination as a coping style in response to dysphoric mood and personality traits like impulsivity, perfectionism, self efficacy expectations, locus of control and self-esteem. In this context we expect MediMind to have a positive effect on satisfaction with life of the participating medical students. Additionally, a measure of mindfulness will be included to assess the expected improvement in mindfulness by participation in MediMind.

## Methods/Design

### Trial design

**Figure 1:**
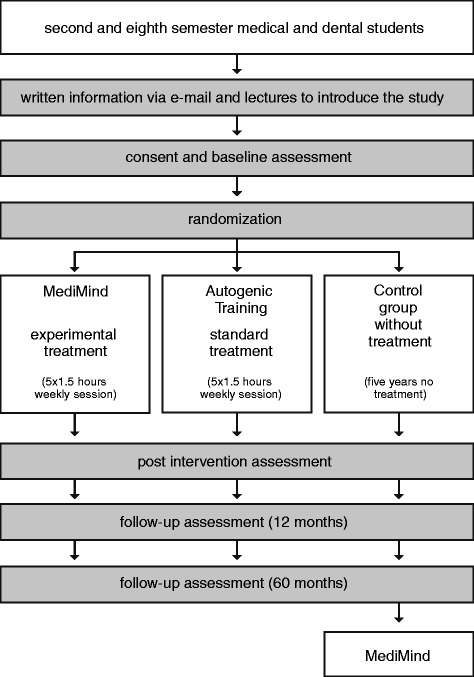
‘**Study design’.**

This is a prospective, randomized, controlled trial, involving four assessment time points (baseline, post-intervention, one-year follow-up and five-year follow-up) and three groups (experimental treatment, standard treatment and control without treatment). Participants are a self-selected group that will be assigned randomly to either the experimental, standard or control group. The longitudinal design will allow the evaluation of the short- and long-term effects of the intervention. Figure 1 shows the overall design of this project. All study procedures, written information and consent forms received approval from the local ethics committee (Landesärztekammer Rheinland-Pfalz, file number: 837.380.13/9065-F) and the University Medical Center data protection official. To report this randomized controlled trial we made use of the CONSORT guidelines [52,53].

### Participants and recruitment

Participants are medical students in the second and eighth semester. In addition to students of human medicine in the preclinical cohort, dental students will be included. Their curriculum and learning environment is nearly analogous to medical students in the preclinical semesters. Students that fit these eligibility criteria and study at the Johannes Gutenberg-University Mainz form a cohort of approximately 420 students in each academic term (second semester: 50 dental and 200 medical students; eighth semester: 170 medical students). It is expected to recruit a minimum of 30% of the possible participants according to literature [17] each semester resulting in an initial sample size of 126 participants. Over the course of time between baseline and five-year follow-up we expect a maximum dropout rate of 50% leading to a final sample size of 189 participants with participants of 3 semesters. The optimal sample size was calculated through analyzing and integrating related publications [16,17,21] via effect sizes (F-values respective η-square-values, Cohen’s d and Λ for multivariate approaches) with G*Power [47]. A synopsis of needed sample sizes for the different articles and different outcome measures within the articles was constructed and evaluated. This resulted in an approximate value of the minimum total sample size of 126 participants at five-year follow-up for valid analysis of secondary outcome.

Students are informed about the project via e-mail, and the Department of Medicine student body shares information on their social network. Additionally, our team introduces the study design and the opportunity for participation at lectures. At that time, we hand out written information and informed consent. Students who return the signed informed consent are introduced to the baseline assessment. They have the choice of either filling out the questionnaire as a paper-and-pencil version or completing an online version. In addition to the signed informed consent, the completion of the baseline assessment is a requirement of becoming assigned to the study groups.

### Randomization

Following baseline assessment, the participants are allocated to one of the three conditions: MediMind, Autogenic Training or control group. Participants with informed consent are stratified randomized initially by course of study (medical versus dental), semester (2nd or 8th) and gender. This was due to control for potential confounding effects and secure homogenous distribution among groups. The randomization to the groups will be with an allocation ratio 2 (MediMind) : 2 (Autogenic Training) : 1 (control group), for ensuring a maximum power for analysis between MediMind and Autogenic Training. After stratification the groups are randomized by drawing lots using the aforementioned allocation ratio. The randomization to the groups will be done by an independent member of institute not involved in this project, ensuring that trainers and recruiters are kept blind to the allocation of each participant. Treatment participants are openly informed into which group they are allocated. Additionally, participants in the control group will be informed of their status and the opportunity to attend MediMind when data collection is completed after five years. Over these five years participants of the control group get no intervention but are invited to fill out the questionnaires at each of the four assessment time points.

### Trainers

Interventions will be presented by four trainers who are qualified and skilled in imparting the manualized contents of the experimental and standard treatment group to students. Two clinical psychologists with additional training in psychotherapy and experience in leading group psychotherapy are chosen for the experimental group (MediMind). They have personal experience in mindfulness meditation and attended external training. They are advised in conducting the training and all details concerning the implementation of the training are available in a comprehensive script. The standard treatment group (Autogenic Training) is presented by two trainers: a sports scientist certified as a trainer of Autogenic Training with long-term experience in teaching these techniques to students and a clinical psychologist with additional training in psychotherapy and Autogenic Training. The treatment of Autogenic Training is also based on a comprehensive script.

### Intervention

Both treatment groups meet over a period of five weeks with a weekly session of 1.5 hours. Within both groups further information and assignments concerning the topics of each training session are provided by an accompanying booklet or handouts.

Based on experiences with a pilot run of the intervention training (MediMind), there should not be more than 15 participants in each training group.

### Mindfulness-based stress prevention training for medical students (MediMind)

Each meeting begins with various types of mindfulness meditation being practiced and a reflection on the assignments of the last session. The specific contents of the training sessions are described below. With every coping skill we teach our participants, we establish a connection to the idea of mindfulness.

Module I: In the first session, the participants get to know each other and are introduced to the themes ‘mindfulness’ and ‘experiencing stress’. Initially, we use short interaction exercises to connect the participants. Following this, based on actual studies on stress experience and psychological morbidity, the relevance of a stress prevention training for medical students is demonstrated. Subsequently, the participants reflect on their own everyday stressors. Afterwards, the methods of MediMind are presented as effective in dealing with stress experience. This is followed by a brief introduction to the history and mode of action of mindfulness. Using a mindfulness exercise, the principle is experienced and made concrete. A practice assignment allows participants to try different experiments in mindfulness in their daily lives until the next meeting.

Module II: The second module addresses two basic concepts of mindfulness. First, the image of the ‘satellite-position’ as a target state of successful stress management is introduced. It characterizes the ability to observe one’s thoughts, emotions, physical reactions and impulse to act. In exercises and real-life examples, participants learn the importance of a presence-of- mind attitude in order to realize and target stress constructively. In addition, participants learn to address intrusive and distracting thoughts or feelings. Through exercises, the participants learn how an accepting attitude can influence the reduction in these thoughts or feelings and release them. By this approach the dysfunctional effect of displacing or avoiding the experience of stress is made clear. In one assignment, participants are asked to log their own level of stress over the week prior to the next meeting, and practice different experiments to switch into the ‘satellite-position’.

Module III: The manner in which cognitive judgment mechanisms influence one’s own experience is imparted to the participants. For a better understanding, we use a stress induction exercise and study everyday examples in order to derive how one’s own appraisal can have a stress heightening influence. These dysfunctional cognitive judgement mechanisms (errors in reasoning) are discussed by the participants in small groups and then presented to the group. Prior to the next session, an assignment will enable the participants to identify errors in reasoning and practice the use of functional reevaluation. An audio CD with a breathing meditation is handed out to the participants to support the practice of mindfulness meditation.

Module IV: This session deals with personal standards and assumptions that have a causal influence on cognitive judgment mechanisms and thus may exacerbate the experience of stress. With the help of everyday life examples, the idea of ‘stress exacerbating settings’ is conveyed to the participants and they can test themselves to determine their core beliefs. It will then be discussed as to which of these core beliefs exacerbate the experience of stress and which can also be helpful. An educational film that emphasizes some specific assumptions in medical education is used to question them critically. Another focus is to highlight how the stress heightening effect of individual core beliefs can be mitigated. In an exercise participants practice coping with functional stress exacerbating settings in the form of various experiments that are grounded on both mindfulness-based and cognitive behavioral therapy.

Module V: The last module focuses on stress-tolerance skills and the concept of radical acceptance [23]. First, the participants determine the characteristics of individual high-tension situations, from which the necessity for and effectiveness of stress tolerance skills is derived. These are then presented to the group and experienced through exercises. As a last resort, to cope with tense situations that can neither be influenced by their external circumstances nor by stress-tolerance skills, the concept of radical acceptance is developed and applied in a practical exercise. At the end of the training, participants draw conclusions and compile worksheets defining what they learned and what they want to remember in the future.

### Autogenic training

The participants learn basic skills of Autogenic Training according to the Schultz method [54]. This is an auto-suggestive relaxation technique in which participants are first instructed by a qualified coach and subsequently able to instruct themselves. These instructions consist of six exercises with corresponding formulas that are subvocally repeated (e.g. ‘My arm is very heavy’) and suggest specific autonomic sensations: muscular relaxation, vascular dilation, stabilization of heart function, regulation of breathing, regulation of visceral organs and regulation of blood flow in the head [55]. Each session contains a theoretical introduction to the practice, the performance of relaxation techniques and a final discussion. The training will be extended by additional exercises including progressive muscle relaxation, breathing relaxation, exercises for body awareness, imaginary journeys and qigong movements. The participants are provided with information material for individual practice.

### Outcome measures

#### Assessment time points

Measures will be completed by all participants at four assessment time points: (1) baseline, after receiving signed informed consent and before random assignment to the study groups; (2) post-intervention, three weeks after the last training session; (3) follow-up, one year after post-intervention assessment; (4) follow-up, five years after post-intervention assessment. At each assessment time point, participants complete the same questionnaires and standard demographic measures, which take approximately 45 minutes. Additionally, starting with post-intervention, questions relating to stressful life events will be added. The participants will be able to complete the baseline questionnaires after the information lectures or complete an online version. In order to participate in the post-intervention and follow-up assessments, the participants will have the choice of either filling in the questionnaire as a paper-and-pencil version or completing an online version. As an acknowledgment of their participation, 50.- € vouchers will be raffled among the participants. As a special motivation to the participants that have been allocated to the control group, everyone who participated in the questionnaire surveys will receive a 20.- € voucher.

#### Primary outcome measure

*Trier Inventory for the Assessment of Chronic Stress (TICS)* [50]. This measure is used to evaluate different aspects of chronic stress. It consists of 57 items, which participants are asked to answer on a Likert-type scale ranging from 0 (‘I never experienced this’) to 4 (‘I experienced this very often’) as to how often the described stressful situations were experienced during the past three months. Based on nine subscales it assesses ‘work overload’, ‘social overload’, ‘excessive demands from work’, ‘lack of social recognition’, ‘work discontent’, ‘social tension’, ‘pressure to perform, ‘social isolation’ and ‘chronic worrying’. The measure presents good to excellent results in terms of internal consistency with Cronbach’s α ranging from .84 to .91. A nine-factor solution of a principal component analysis confirms a good construct validity and correlations between the TICS and other stress questionnaires show plausible relationships [50].

#### Co-primary outcome measure

*Brief COPE* [56]. The Brief COPE includes 28 items which measure 14 conceptually differentiable coping reactions. Each scale is measured by two items and comprises effective and ineffective coping strategies such as ‘self-distraction’, ‘active coping’, ‘denial’, ‘substance use’, ‘use of emotional support’, ‘use of instrumental support’, ‘behavioral disengagement’, venting’, positive reframing’, ‘planning’, ‘humor’, ‘acceptance’, ‘religion’ and ‘self-blame’. The participants are asked to rate on a Likert-type scale ranging from 1 (‘not at all’) to 4 (‘very much’) how much the statements resemble the person’s thoughts and actions in demanding or difficult situations in the past. Internal consistency reliabilities were found to be poor to good with a range between .50 and .90 (Cronbach’s α). An exploratory factor analysis provides a nine-factor solution which was similar to results reported for the full inventory [56]. Information about the convergent and discriminant validity is given for the previously published full inventory [57]. Correlations between functional coping strategies and personality qualities regarded as beneficial are reported. Furthermore, the COPE scales were unrelated to a measure which is different to the coping styles assessed in the COPE inventory. A German version of the Brief COPE is available and has been validated [58]. Due to a lack of internal consistency and item variance, the authors of the German translation recommend the selection of 11 subscales which are subsumed under four factors of coping. This solution presents acceptable to good internal consistency with Cronbach’s α ranging from .61 up to .81 [58].

### Secondary outcome measure

*Brief Symptom Inventory (BSI)* [59]. The BSI is a 53-item measure that focuses on impairment due to somatic and psychological symptoms. Based on nine subscales it comprises ‘Obsessive-Compulsive’, ‘Paranoid Ideation’, ‘Hostility’, ‘Somatization’, ‘Depression’, ‘Interpersonal Sensitivity’, ‘Anxiety’, ‘Psychoticism’ and ‘Phobic Anxiety’. On a Likert-type scale ranging from 0 (‘not at all’) to 4 (‘extremely’) the current distress is to be indicated. Reliability estimates for the German version were found to be good in a community sample with Cronbach’s α ranging from .70 to .88. Correlations between the BSI and other instruments show plausible relationships [60].

### Additional measures

*Response Style Questionnaire (RSQ)* [61]*.* In its short version, the RSQ is a 23-item questionnaire of cognitive and behavioral coping styles in response to dysphoric mood. It comprises the three scales ‘symptom-focused rumination’, ‘self-focused rumination’ and ‘distraction’ for which participants are asked to indicate their normal behavior when feeling sad or depressed on a Likert-type scale ranging from 1 (‘almost never’) to 4 (‘almost always’). Psychometric properties referring to the German short version including Cronbach’s α ranging from .75 and .88 were found to be acceptable for a depressed inpatient sample and a community sample. A principle component analysis confirmed the subscales with a three-factor solution. Moreover, convergent and discriminant validity have been confirmed by plausible associations with related and unrelated cognitive constructs [62].

*Skala Impulsives-Verhalten-8 (I-8)* [63]. The I-8 is a German questionnaire that measures impulsive behavior depending on four subscales: ‘immediacy’, ‘purpose’, ‘persistence’ and ‘risk-taking’. These are assessed with two items on a Likert-type scale ranging from 1 (‘doesn’t apply at all’) to 5 (‘applies completely’) referring to how much the statements apply to the participants behavior. Reliability estimates were found to be acceptable to excellent by using McDonald’s omega coefficient ranging from .65 to .92. Due to a confirmatory factor analysis the four factors could be validated. Verification of the construct validity presented good results in terms of plausible associations with related and unrelated cognitive constructs [63].

*Frost Multidimensional Perfectionism Scale (FMPS)* [64]. The FMPS measures different dimensions of perfectionism. According to a differentiated analysis of publications using the FMPS, the author of the German translation recommends a subsumption of the original six subscales into ‘Concerns over Mistakes and Doubts’, ‘Parental Expectations and Criticism’, ‘Personal Standards’ and ‘Organization’ [65]. It consists of 35 items which participants are asked to answer on a Likert-type scale ranging from 1 (‘strongly disagree) to 5 (‘strongly agree’). In the present study only, the subscales ‘Concern over Mistakes and Doubts’ and ‘Personal Standards’ will be applied. Concerning the two selected subscales, this measure presents good results in terms of internal consistency with Cronbach’s α ranging from .78 to .88. Based on the confirmation of the four-factor solution the factorial validity is assessed to be good [65]. In terms of construct validity the new subscales show the same significant relationships with other measures as the original scales [65].

*Freiburg Mindfulness Inventory (FMI)* [66]. The FMI is a German questionnaire that assesses the various attributes of mindfulness. In its short version it comprises 14 items which the participants answer on a Likert-type scale ranging from 1 (‘almost never’) to 4 (‘almost always’) concerning the frequency of reported experience during the past four weeks. Compared to the original instrument, the short form appeared to be easier to answer for subjects without previous meditation experience [67]. The measure presents good results in terms of internal consistency with Cronbach’s α = .86. A one-factor solution of a principal component analysis and correlations between the FMI and other relevant constructs like self-awareness, meditation experience and dissociation confirm a good construct validity [67].

*Short Scale for Measuring General Self-efficacy Beliefs (ASKU)* [68]. The ASKU is a German three-item questionnaire to assess general self-efficacy expectations. Appraisal of one’s own competencies of planning and executing actions in a successful way is given on a Likert-type scale ranging from 1 (‘doesn’t apply at all’) to 5 (‘applies completely’). Data about good internal consistency range from .81 to .86 (McDonald’s ω). Due to a confirmatory factor analysis, the tested model of a one-factor solution could be validated. Verification of the construct validity presented good results in terms of correlations with other measures of self-efficacy expectations and related constructs [68].

*Satisfaction with Life Scale (SWLS)* [69]. The SWLS is a 5-item assessment of life satisfaction. On a Likert-type scale ranging from 1 (‘strongly agree’) to 7 (‘strongly disagree’), respondents indicate the extent to which they agree with each statement. The German version of the measure has good psychometric properties. Reliability estimates in a general population sample were found to be excellent in terms of Cronbach’s α = .92. Evidence of convergent validity is given by plausible relationships between the SWLS and measures of depressiveness and social support [70].

*Skala Internale-Externale-Kontrollüberzeugung (IE-4)* [71]. The IE-4 is a German questionnaire for the assessment of locus of control and comprises the two subscales ‘internal locus of control’ and ‘external locus of control’. Each subscale consists of two items for which the respondents are asked to indicate on a Likert-type scale ranging from 1 (‘doesn’t apply at all’) to 5 (‘applies completely’) how much the statements apply to their own conviction. The measure presents poor to good results in terms of internal consistency with McDonald’s omega coefficient ranging from .53 to .71 in a general population sample. Due to a confirmatory factor analysis, the two factors could be validated. Positive correlations between the subscale ‘internal locus of control’ and general self-efficacy beliefs or optimism confirm a good construct validity. This statistical relationship is exactly reverse concerning the subscale ‘external locus of control’. An additional evidence of convergent validity shows the high association between the IE-4 and an alternative measure of the same construct [71].

*Rosenberg’s Self-Esteem Scale (RSS)* [72]: The RSS is a 10-item questionnaire that assesses global self-esteem. It has been translated into the German language [73] and partially revised by excluding one item that was determined to be psychometrically weak [74]. On a Likert-type scale ranging from 0 (‘strongly agree’) to 3 (‘strongly disagree’), respondents indicate the extent to which they agree with each statement. The evaluation of the revised version presents good results in terms of internal consistency with Cronbach’s ranging from .84 to .85. A one-factor solution of a principal component analysis confirms a good construct validity [74]. Information about the convergent validity of the unrevised version is given by correlations between the RSS and measures of self-efficacy expectations and optimism [73].

### Statistical methods

Descriptive statistics on study population will be calculated for each group at baseline. Means and standard deviations on primary, co-primary, secondary outcome and additional measures will be presented at baseline, post intervention and at the two follow-up time points for each group. A two-folded approach according to Tabachnik and Fidell [75] is used to address missing data. Initially, a Completer-Analysis with a sample of complete data-sets of each person is evaluated. Secondly, an Intent-to-Treat-Analysis with a Last-Observation-Carried-Forward-Method will be applied to check for differences and possible drop-out-effects. The null hypothesis will be rejected at the cut-off of 5% (α-level), therefore a *P* value ≤ .05 will be applied. To evaluate all group x time interactions in one model, we will use an explicatory MANOVA with primary, co-primary and secondary outcome. We expect differential time x group effects on secondary outcome at the one year/five year follow-up after post-intervention assessment. Therefore, this preliminary analysis will be called ‘explicatory’. For confirmatory analysis following the assumption that primary and co-primary outcome are separate hypothesis, both hypotheses will be tested in separate repeated-measures ANOVA’s with the factorial structure group (MediMind, Autogenic Training and control group) x time. The hypothesis will be evaluated through testing interaction effects. Orthogonally constructed comparisons are then used to check for pairwise differences. If needed, Holm-Bonferroni corrected pairwise comparisons will be used [76]. Additionally post-hoc sub-group comparisons (sex, semester, cohort) will be conducted to analyze data sets furthermore.

### Effect size

To quantify the effects, partial η-square values will be calculated using cut-off-norms provided by Cohen [77]. In advantage to Cohen’s d, the amount of differential effect in the interaction model can be quantified. Subgroup differences will also be quantified using partial η-square values.

All statistical analyses will be conducted using either SPSS version 21 (SPSS Inc., Chicago, IL, USA) or Microsoft Office Excel (Microsoft Corporation, Redmond, WA, USA)

## Discussion

Our study is the first to evaluate the effectiveness of a mindfulness-based intervention for medical students in Germany in a randomized controlled setting with both treatment groups, MediMind and Autogenic Training, and a control group without treatment. With the possibility of controlling several confounding aspects, the analysis will be based on an experimental design with good power.

One possible limitation of the study might be a motivational bias of the selected participants. It can be assumed that mainly highly motivated students volunteer to take part in a weekly training with an additional time requirement beside the high workload of their curriculum. Possibilities to generalize the results may be limited to students of high motivation and interest in the concept of mindfulness and Autogenic Training. Furthermore, the response rate at the time of post-intervention might be affected as the data cannot be collected directly at the end of the last training session. The students will be asked to send back a paper-and-pencil version of the questionnaire or to complete an online version. Considering the fact that participants of the control group do not benefit from the training until the data collection is completed after five years, the motivation of this specific study group to complete the questionnaires at post-evaluation is expected to be challenging. Further drop-outs could occur one year or five years post-intervention, as participants will be harder to reach or no longer motivated. In order to minimize a reduction in sample size, monetary vouchers will be raffled among the participants. Another limiting effect might be the inclusion of students at different stages of their studies. The experience of stress might differ between the second and eighth semester, and therefore, the effect size of a pre-post effect of the intervention is supposed to be smaller in the advanced semester.

In spite of possible limitations, this study will be one of the few attempts to explore the possibility of stress prevention in a German medical school. In order to address limitations of previous research and to correspond to the demands called for in reviews of the literature [13-15], we use a rigorous study design. Therefore, a strength of our project is the three-group design and the randomized assignment of participants to treatment and control groups. Taking into account the capabilities of implementing a stress prevention training in the medical curriculum, it seems to be more realistic to anticipate a voluntary participation. In that case, our study design reflects a naturalistic setting. Follow-up assessments and the use of validated outcome measures will enable us to ascertain the prevention effect of our intervention. Moreover, the inclusion of students of the second and eighth semester will allow us to compare the impact of intervention at different stages of medical training. By exploring personality traits assumed to work as specific risk factors in medical training, we hope to point out whether a stress prevention training like MediMind will be able to affect them positively. Additionally, these measurements may identify moderator variables to determine which intervention works best for whom.

## Trial status

Recruitment for this project started in November 2013 and is ongoing until at least the initial minimum sample size with regard to the expected dropout rate is reached.

## Abbreviations

ANOVA: analysis of variance
ASKU: Short Scale for Measuring General Self-efficacy Beliefs
BSI: Brief Symptom Inventory
FMI: Freiburg Mindfulness Inventory
FMPS: Frost Multidimensional Perfectionism Scale
IE-4: Skala internale-externale-kontrollüberzeugung
I-8: Skala Impulsives-Verhalten-8
MANOVA: multivariate analysis of variance
MediMind: Mindfulness-based Stress Prevention Training for Medical Students
RSQ: Response Style Questionnaire
RSS: Rosenberg’s Self-esteem Scale
RSQ: Response Style Questionnaire
SWLS: Satisfaction with Life Scale
TICS: Trier Inventory for the Assessment of Chronic Stress
